# Facile Transfer of Spray-Coated Ultrathin AgNWs Composite onto the Skin for Electrophysiological Sensors

**DOI:** 10.3390/nano13172467

**Published:** 2023-08-31

**Authors:** Minwoo Lee, Jaeseong Kim, Myat Thet Khine, Sunkook Kim, Srinivas Gandla

**Affiliations:** Multifunctional Nano Bio Electronics Lab, Department of Advanced Materials Science and Engineering, Sungkyunkwan University, Suwon 16419, Republic of Korea; alchemist@skku.edu (M.L.); j2castle@naver.com (J.K.); myatthetkhine0997@gmail.com (M.T.K.)

**Keywords:** spray coating, AgNWs, glossy paper, conformable, electrophysiological, sensors

## Abstract

Disposable wearable sensors that ultrathin and conformable to the skin are of significant interest as affordable and easy-to-use devices for short-term recording. This study presents a facile and low-cost method for transferring spray-coated silver nanowire (AgNW) composite films onto human skin using glossy paper (GP) and liquid bandages (LB). Due to the moderately hydrophobic and rough surface of the GP, the ultrathin AgNWs composite film (~200 nm) was easily transferred onto human skin. The AgNW composite films conformally attached to the skin when applied with a LB, resulting in the stable and continuous recording of wearable electrophysiological signals, including electromyogram (EMG), electrocardiogram (ECG), and electrooculogram (EOG). The volatile LB, deposited on the skin via spray coating, promoted rapid adhesion of the transferred AgNW composite films, ensuring stability to the AgNWs in external environments. The AgNWs composite supported with the LB film exhibited high water vapor breathability (~28 gm^−2^h^−1^), which can avoid the accumulation of sweat at the skin–sensor interface. This approach facilitates the creation of rapid, low-cost, and disposable tattoo-like sensors that are practical for extended use.

## 1. Introduction

The assessment of pathological health conditions through the non-invasive monitoring of electrophysiological (EP) signals using epidermal electronic sensors is of significant interest, as it enables the early detection of abnormal physiological activities and prevents the development of chronic diseases [1]. The field of “epidermal electronics” [2] has made significant progress in developing new functional materials and processing techniques to achieve skin-conformable devices and sensors. As a result, sensors are becoming thinner and more flexible than ever to meet skin-compliant electronics requirements [3,4,5,6,7].

Epidermal electronic devices are designed for the development of human–machine interactions (HMIs) controlled by EP signals, including EMG and EOG [8,9,10]. The electrodes used in clinical and research settings for EP signal recording are predominantly gel and dry electrodes [11,12]. However, the dehydration of gel electrodes over time greatly limits their long-term monitoring utility. Thus, dry electrodes have emerged as a promising alternative to gel electrodes [13,14]. Considerable research efforts have been dedicated to developing various types of dry electrodes that can enhance signal quality, including PEDOT: PSS/graphene [15], natural silk protein/CNT [16], PEDOT: PSS/WPU/D-sorbitol [17], Au/Cr/PI/SiO2/PDMS [18], polyamide-based nanofibers and AgNWs [19], Ecoflex mixed with silver microparticles [20], and graphene/PMMA [21]. However, materials based on AgNWs have gained increasing attention due to their promising attributes and applications [22,23,24]. These electrodes can be attached to the skin using either a supportive stretchy band or a sticky substance, with the latter gaining more attention due to their self-adhesiveness and excellent conformability to the skin. To achieve a conformable skin–electrode interface, the electrode film should have a low thickness (in the nm–µm range) or Young’s modulus between 0.1 and 1000 kPa. However, for wearable electronics, it is crucial to use inexpensive materials and keep fabrication costs low. While some skin-like breathable and conformable sensors have been reported, their materials are often too costly to be practical. On the other hand, temporary tattoo dry electrodes have emerged as a promising technology in the field of epidermal electronics due to their low cost, thin-film form, and long-term wearability on the skin [25,26]. These electrodes are held in place by sticky substances, as first introduced by Rogers’ group [27], while tattoo-based sensors were developed by Wang’s group [28]. Commercially available temporary tattoo papers with sub-µm thick transferrable polymer film have been used for laminating sensors to the skin for conformability [1]. However, most of these transferring processes typically involve the use of a water-soluble or releasing layer to facilitate the transfer of functional materials onto the skin.

In this study, we present a simple and cost-effective process for transferring AgNW composite film onto the skin using GP, without the need for a water-soluble or releasing film. We conducted various material and electrical characterizations to determine the necessary information and parameters for LB-supported AgNW composite films. These ultrathin AgNW composite films were successfully used to acquire electrophysiological signals, such as EMG, ECG, and EOG. The transferred AgNW composite films exhibited stable responses to external physical disturbances and remained intact without any significant damage to the surrounding environment. The AgNWs composite processed on GP resulted in a highly transferable, ultrathin, and flexible film that could potentially be extended to other functional materials for the development of ultraconformable wearable electronics.

## 2. Materials and Methods

### 2.1. Materials

A commercial AgNW composite solution was purchased from Flexioink, SG FLEXIO Co., Ltd. (Daejeon, Republic of Korea) at the price of approximately USD 240 for 500 g. The cost may be high; however, it can be significantly reduced by purchasing in bulk. The solution was stored in a refrigerator (2–8 °C) and remained stable for up to a year. The solution provided by the manufacturer contains AgNWs and hydroxypropyl methylcellulose (HPMC). The AgNWs are approximately 21 μm in length and have a diameter of ~26 nm. Both AgNWs and HPMC were dispersed in a DI water solvent. A LB from 3M Nexcare^®^, 3M (Maplewood, MN, USA) was purchased and used without any further modification. The GP used, a polyethylene-coated paper film with an approximate roughness of 3.8 ± 0.5, was procured from Sungji Company, (Hwaseong, Republic of Korea).

### 2.2. Film Preparations and Transfer to the Skin

The AgNW composite solution was spray-coated onto a GP surface supported by a glass substrate using a commercial spray gun (Hi-Line HP-CH, Iwata, Japan). The optimization of the AgNW composite deposition involved the following parameters: a sample spray-coating distance of 20 cm, a spray-coating pressure of 0.1 MPa, and a total of 4 spray-coating rounds—2 horizontally moving downward and 2 vertically moving to the right. The entire process was conducted by placing the sample on a preheated (90 °C) hot plate. The resulting AgNW composite film was baked for at least 20 min before use. To transfer the AgNW composite film onto the skin, 3M Nexcare^®^ liquid was initially sprayed onto the forearm skin. The GP holding the AgNW composite film, with the AgNWs composite facing the sprayed LB surface, was then laminated onto the skin. Finally, the GP was peeled off after a few seconds, leaving only the AgNW composite film successfully transferred onto the skin.

### 2.3. Film Characterization

Water vapor permeability: To determine the water vapor permeability (WVP) of the test samples, we followed the specifications outlined by McHugh et al. [29]. The test cell comprised a glass vial filled with 10 mL of water. To measure the WVP, the AgNW composite film supported by the liquid bandage was transferred and positioned across the opening of the vial. The weight loss of the cell was measured over a period of 30 days, and the WVP was calculated using ASTM-E 96 (ASTM, 1995b) standards [30,31]. Two samples were tested for this test.

Contact angle: Contact angle measurements were conducted using a SMARTDROP (Femtobiomed Co., Ltd., Seongnam, Republic of Korea) at an ambient temperature of 25 °C and relative humidity of 40%. A 5 μL liquid droplet was dispensed from a syringe onto the test sample. The syringe was then withdrawn, and the static contact angle was imaged using a charge-coupled device (CCD) camera twice within 5 s and once after 60 s. The measurements were conducted on two samples in each case, with three points taken from each sample.

Optical transmittance: The transmittance spectrum collected in the range of 400~800 nm was obtained using a UV–vis spectrophotometer (V-770, JASCO, Tokyo, Japan). Three samples were tested for this test. 

## 3. Results and Discussion

**Figure 1:**
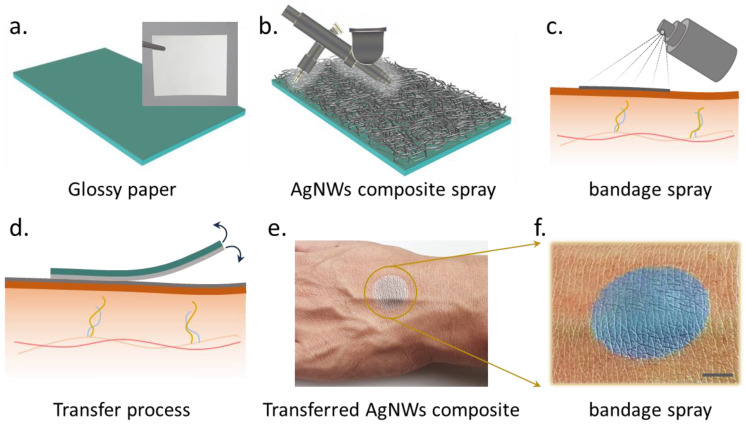
The wearable sensor fabrication process (**a**–**f**) involves transfer onto the skin. First, a water-based AgNW composite solution was spray-coated onto tattoo paper and then preheated on a hotplate at 90 °C for 20 min. Additionally, the LB was sprayed onto the skin. Subsequently, the tattoo paper containing the AgNW composite was laminated onto the skin with the AgNWs composite facing the skin, resulting in ultraconformable AgNW–based electrodes (scale bar: 5 mm).

**Figure 2:**
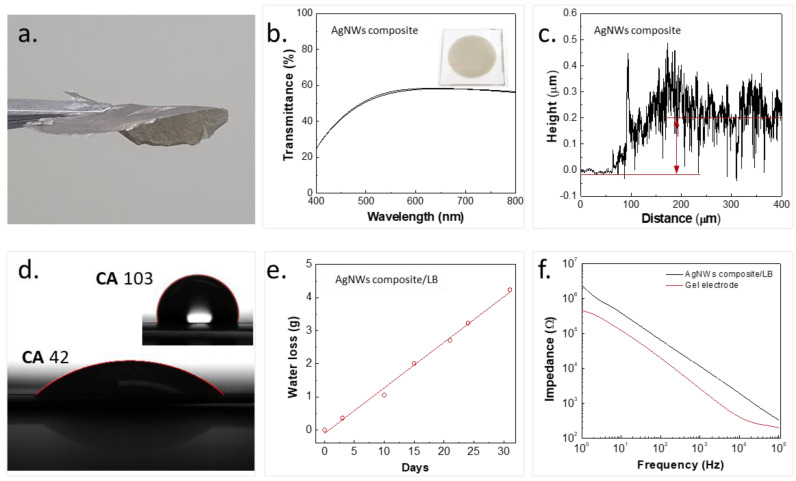
The AgNW composite sensor electrode and its characteristics. (**a**) LB–supported floating AgNW composite electrode. (**b**) Transmittance spectrum, (**c**) thickness profile, and (**d**) contact angles of water on spray–coated AgNW composite and LB surfaces on GP. (**e**) Water loss over time of AgNW composite/LB film (same as shown in Figure 2a): the film covered the opening of a vial to determine the water vapor permeability. (**f**) Electrical impedance of AgNW–based and conventional electrodes.

**Figure 3:**
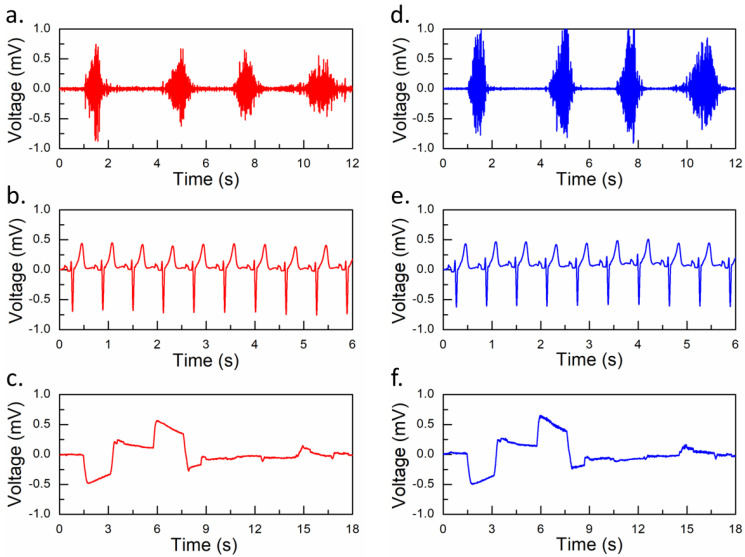
Electrophysiological signals (EMG, ECG, and EOG) recorded using (**a**–**c**) conventional and (**d**–**f**) AgNW–based electrodes.

The process of transferring a spray-coated ultrathin AgNW composite film on GP onto human skin using a biocompatible LB spray is illustrated in Figure 1a–f. A comparison of our work with previously reported research is provided in Table S1 [32,33,34,35,36,37,38]. A circular shadow mask, prepared using a UV nanosecond laser, was used for depositing the AgNW composite by spray-coating. Figure 1a shows a schematic illustration of the GP with an inset showing the real image. Due to the highly volatile nature of the LB spray solution, the solution sprayed on the forearm skin readily formed an ultrathin sticky layer. The liquid spray is commercially available, and the LB forms a layer that is biocompatible as per the product details. Once the sticky layer is formed, the AgNW composite film on GP is immediately transferred, and after a few seconds, the GP is removed, leaving a conformable AgNW composite film on the skin (blue color). The transfer of the AgNW composite to the Tegaderm™ tape is shown in Figure S1. Since Tegaderm™ tapes are currently being used in the medical field, it is important that the AgNW composite is transferable to them. As seen in the figures, the AgNW composite spray-coated on GP can be easily transferred without any failure. The floating film of the AgNW composite with a LB is shown in Figure 2a. Its mechanical tensile stress versus strain behavior is depicted in Figure S2. The films exhibited stretchability up to ~25%. SEM images of the spray-coated AgNW composite are shown in Figure S3a–c. Figure S3d illustrates the distribution depicting the relationship between the diameter of the AgNWs and their corresponding number. The AgNWs exhibited an average diameter of approximately 26 nm. The films composed of the AgNW composite and the LB, arranged in layers, demonstrated approximately 60% transparency in the wavelength range of 550 to 800 nm. (Figure 3b). The thickness of the AgNW composite films was measured using laser scanning microscopy (OLS5100-LAF laser microscope, EVIDENT Corp., Tokyo, Japan) and was found to be ~200 ± 55 nm for the two samples (Figure 2c). The measured contact angles (CAs) of DI water droplets on the AgNW composite and LB surfaces on GP were ~42° ± 4° and ~103° ± 3° (Figure 2d), respectively, while on GP, it was ~76° ± 4° (Figure S4). Due to the low adhesion of water to the LB film, the LB film exhibited high CA. This property can be useful for handling in a water environment, as discussed in the environmental stability section. The conformability and signal-to-noise ratio (SNR) of the AgNW–based films are crucial for its use as a wearable sensor for EP monitoring. The larger the contact area between the skin and the sensor film, the higher the conformability, and thus, the lower the impedance and higher the SNR [9,10]. The theoretical analyses have proven that films with thicknesses of around a few micrometers mimic the wrinkles of the skin with high conformability [39]. Therefore, the conformability, thickness, impedance, and SNR are correlated with each other, as discussed in more detail in the following sections.

### 3.1. Water Vapor Permeability or Breathability

The WVP evaluation was carried out by measuring the weight loss of water over time, as stated in Section 2. The ability of a film to allow sweat vapors to pass through depends on its WVP. The higher the WVP of a film, the more it allows sweat vapors to permeate. For the stable recording of signals, the sensor should not only require conformal contacts with sufficient adhesion to the skin but also WVP greater than that of the skin (8–20 gm^−2^h^−1^) to avoid the accumulation of sweat, which affects the signal quality. The AgNW composite films supported with LB films demonstrated high WVP of approximately 28 ± 2 gm^−2^h^−1^ in two samples (Figure 2e), primarily due to the ultrathin film forms. Other potential mechanisms contributing to the water vapor permeability (WVP) could include sorption–diffusion through the polymer matrix, nano-level porosity enabling the movement of water vapor molecules, and the material’s rough morphology. Additionally, the hydrophilic nature of HPMS, a polymer with an affinity for water, could lead to water vapor diffusion driven by concentration gradients.

### 3.2. Skin–Electrode Contact Impedance

The quality of electrophysiological signals recorded depends greatly on the interface between the electrode and the skin surface. Skin–electrode contact impedance can significantly impact the SNR ratio, which in turn affects the quality of the signals recorded. Typically, low conformal contact between the electrode and the skin results in high background noise and a lower SNR. Therefore, reducing the impedance is crucial in the application of EMG sensors. To qualitatively measure conformal contact, electrochemical impedance spectroscopy was employed. The evaluation setup involved two electrodes (positive and negative) laminated on the brachioradialis muscle of the forearm, separated by a distance of 5 cm, and a ground electrode laminated on an area with less muscle or an irrelevant muscle, such as the backside of the palm. The electrodes had the same shape and area (20 mm diameter). The impedance of both the AgNW–based and conventional gel electrodes was measured using the IVIUMnSTAT (multichannel electrochemical analyzer) in the frequency range of 1 Hz to 100 kHz (as shown in Figure 2f). Both electrodes exhibited a similar descending trend with an increase in frequency. Four impedance measurements were conducted on four distinct sets of AgNW–based electrodes, all exhibiting a consistent trend without any noticeable differences. The AgNW composite film transferred onto the skin exhibited excellent conformability with good adhesion, indicating low skin-electrode interface impedance. However, the AgNW–based electrode displayed a higher impedance than the gel electrode, likely due to the use of carbon tape to connect the AgNW composite film and the measuring instrument electrode. Although the AgNW–based film and skin had conformal contact, the carbon tape adhered to the AgNW composite film possessed less conformability because of their different surface morphologies, leading to gaps at the interface and causing an increase in impedance. At this stage, the microscale gap at the interface is unavoidable, and addressing this issue is a future research goal. 

### 3.3. Electrophysiological Measurements

Electrophysiology is a field that deals with the electrical properties of biological organs and is useful for electrodiagnosis and monitoring. Three electrophysiological measurements—electromyography, electrocardiography, and electrooculography—were carried out to record the electromyogram (EMG), electrocardiogram (ECG), and electrooculogram (EOG). In all cases, bio-potential signals were recorded using a commercially available MP36 data acquisition system (Biopac Inc., Goleta, CA, USA) with a three-electrode configuration (positive, negative, and reference). The sensors generated a bio-potential voltage that was sent to the circuitry, and the output voltage provided a recording of the muscle activity. The system used a differential amplifier, a bandpass filter, and a gain amplifier. Electromyography is a technique that records the electrical activity produced by skeletal muscles using an instrument called an electromyograph. Under neurological activation, the muscle cells of skeletal muscles are electrically activated, giving rise to bio-electric potential.

To evaluate the EP signals acquired from an AgNW–based sensor, it is important to record simultaneously from both conventional gel electrodes (Ag/AgCl, Kendall 135SG) and AgNW–based electrodes for comparison. Here, the AgNW–based film and conventional gel electrodes are termed sensors as they sense the bio-potential signals through these electrodes. The EMG signals for both electrodes, positioned near their counter sensor (polarity) parts, were recorded from the forearm flexor muscle. The sensor electrodes were transferred as described in the Film Preparations and Transfer to the Skin section. One side of the carbon tape was connected to the sensor, and the other end was connected to a cable (for connection to Biopac electrodes) with conductive epoxy. The EMG signals recorded for multiple hand squeeze and release motions of both sensors are shown in Figure 3a,d. The electrode placements on the skin for recording the EMG, ECG, and EOG signals are shown in Figure S5. All signals displayed similar trends, except for the high signal-to-noise of the conventional gel electrodes. The filters used were a bandpass filter of 30–250 Hz and a bandstop filter of 60 Hz. The SNR values of the AgNW–based electrode and the conventional Ag/AgCl electrode were 24.15 dB and 32.73, respectively.

The ECG signal is a graph that measures the heart’s electrical activity in terms of voltage and time. One cycle of the ECG bio-potential signal represents the depolarization and repolarization of the cardiac muscle. Although traditional ECG recording requires ten electrodes placed on the limbs and surface of the chest, ECG signals recorded using only three electrodes can provide basic yet sufficient information. The ECG signals acquired from electrodes laminated on the left (positive) and right (negative) sides of the human chest, and on the lower left abdomen (ground) in the case of both sensors, are shown in Figure 3b,e. The filters used were a high-pass filter of 0.05 Hz and a low-pass filter of 150 Hz with a sampling rate of 2 kHz. The ECG signals recorded with both sensors show a similar trend.

Next, we performed the EOG signal measurement. Electrooculography measures the corneo-retinal potential that exists between the anterior and posterior sides of the eye. A pair of electrodes are placed on either side (left and right) of the eyes with a ground electrode behind the ear. The EOG signal was recorded by moving the eyes right, left, down, and up (Figure 3c,f). The filters used were a bandpass filter of 0.05–35 Hz and a notch filter of 60 Hz to filter out involuntary noise and signals. Overall, both electrodes delivered similar performances, signifying the potential use of AgNW–based electrodes. 

### 3.4. Stability under Physical Disturbances

**Figure 4:**
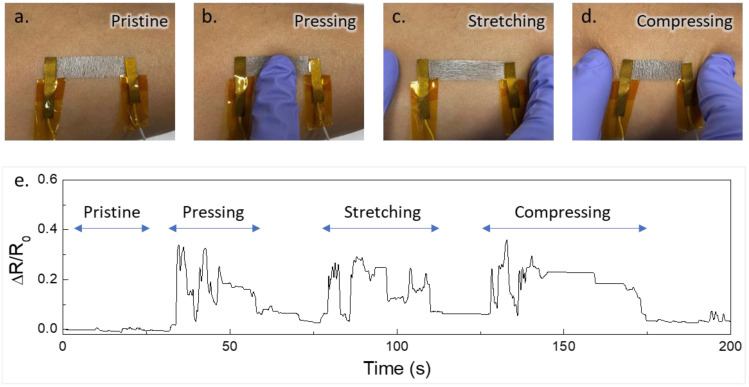
The AgNW–based electrode under external physical movements. (**a**) Pristine, (**b**) pressing, (**c**) stretching, and (**d**) compressing. (**e**) Their corresponding resistance change variations.

The transferred AgNW composite film (45 mm × 10 mm) on the skin subjected to externally applied mechanical stimuli, such as pressing, stretching, and compressing, is shown in Figure 4a–d. Wearable sensors may be prone to external physical disturbances while performing daily activities. Therefore, it is important to examine the performance of the film under mechanical stimuli. Note that extreme applied mechanical force can damage the film; thus, a moderate force was applied (about 1.5 N). The film’s performance was evaluated by forming a closed-loop circuit with two electrodes in contact with either end of the film to monitor the resistance changes caused by the mechanical stimuli. The resistance changes, represented as the relative change in resistance (ΔR/R_0_), were plotted against time (Figure 4e). The increase in resistance can be attributed to two reasons. First, small portions of fracture in the AgNW composite film can cause a slight increase in the mean free path for the electrons to move from one end of the electrode contact to the other end. Second, the film-to-contact electrode interface can be disturbed by any physical movement that disturbs the AgNW composite system. The resistance change was nearly 30% with all physical movements compared to the resistance in the pristine case. 

### 3.5. Environmental Stability and Compatibility

**Figure 5:**
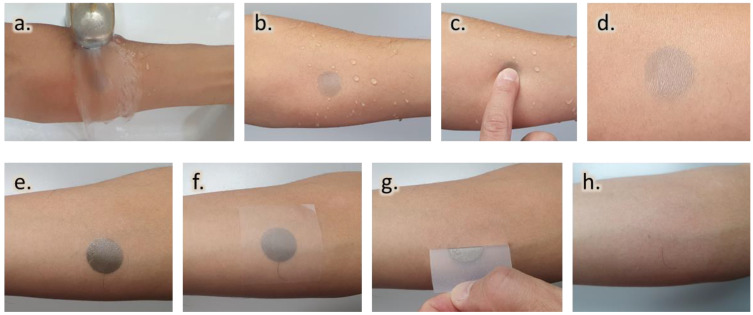
Ultraconformable AgNW–based wearable electrode characteristics. (**a**–**d**) Evaluation of electrode stability in high–humidity external environments under continuous tap water flow: samples during (**a**) and after (**b**) exposure to water flow, and during (**c**) and after (**d**) pressing. Wearable electrode peel–off process: (**e**) initial state, (**f**) application of scotch tape and gentle press for lamination, (**g**) tape removal, and (**h**) skin appearance (without any marks) after tape removal.

For wearable sensors, especially for the continuous monitoring of electrophysiological signals, functional stability is critical. Therefore, it is essential to examine the stability (without material damage) of electrodes. The stability of the film under running tap water was examined as follows: first, subjecting the film to running tap water and second, pressing. The corresponding pictures at each instance are shown in Figure 5a–d. The film’s stable wearability is due to the LB, which is waterproof and will not be affected or removed in water or moisture environments. This has also been confirmed by the contact angle (CA) measurement, which showed high CA to water droplets. Furthermore, Figure S6 presents the recorded EMG signals during fist hand gestures, both before and after exposure to water and pressing. In both scenarios, the EMG signals exhibited remarkable stability, possibly attributed to the presence of an underlying and encapsulated LB film. This film acts as a barrier, offering mechanical stability to the active material even under external water exposure and physical pressure. However, the case of pressing revealed a slight degradation in the signal amplitude. This decrease might be attributed to a minor increase in the electrical resistance of the sensor, potentially caused by microcrack formation. Notably, the applied force for pressing was mild (approximately 1.5 N), as excessive force could lead to the development of microcracks and eventual damage to the AgNW composite film.

Furthermore, the disposal of the AgNW–based film can be carried out by laminating it with commercially available scotch tape (Scotch Magic™ Tape (3M)), which has higher adhesion to the layered LB–AgNW composite film than the film to the skin (Figure 5e–h). Using this procedure, the transferred films could be easily removed, demonstrating that the film is robust, easily removable, and therefore, useful as a wearable sensor. 

The sensor’s long-term stability was evaluated by performing EMG measurements before and after a 36 h period (Figure S7). It is important to note that the EMG signal measurements were conducted within a 2-day timeframe due to the challenges posed by the daily activities involving physical movements, making the handling of the contact pad electrodes with the sensors complex. Therefore, the presence of ultrathin gradient contact pad electrodes is vital for the efficient long-term monitoring of EP signals, complementing the ultrathin attributes of the sensor. The development of robust ultrathin gradient contact pads remains an area open for investigation. Notably, the EMG signals recorded during fist hand gestures before and after the 36 h period exhibited no noticeable differences, underscoring the sensors’ potential for robust long-term use with appropriate contact pad electrodes.

On the other hand, the strict biocompatibility of wearable sensors is controversial and debatable. To address this, a wearable sensor was worn on the forearm for three days. As seen in Figure S8, the wearable sensor before and after being worn for 72 h reveals the absence of any rashes, likely attributed to the presence of a LB (a commercially biocompatible material) interlayer between the AgNW composite and the skin. This interlayer acts as a protective barrier, fostering compatibility between the sensor’s layered structure and the skin, thus ensuring safe prolonged usage. It is worth noting that the primary damage to the sensor after 72 h of usage was observed during showering.

Overall, this work showcases a seamless transfer method for ultrathin AgNW-composite patches onto the skin, eliminating the need for additional materials or complex processes. These sensors not only exhibit resilience against environmental and physical factors but also ensure safe and durable long-term skin monitoring, underlined by their exceptional water vapor permeability.

## 4. Conclusions

The results demonstrate that spray-coated ultrathin AgNW composite transferred using simple and efficient GP in conjunction with a LB presents a promising solution for the efficient transfer of wearable sensors to the skin. The ultrathin AgNW–based sensors delivered stable and continuous EP signals and possessed high water vapor breathability. Other deposition techniques, such as bar-coating, blade-coating, and dip-coating, could also be applied to deposit additional functional materials onto GP for efficient transfer onto the skin. The AgNW composites transferred onto the skin were sufficiently stable to external mechanical stimuli and remained intact. Additionally, the AgNW composite film electrodes on GP can be commercialized so that they can be readily used with a LB transfer. With this study, the approach of achieving conformable sensors by transfer technique can provide new insights into wearable electronics.

## Data Availability

The data presented in this study are available on request from the corresponding author.

## Supplementary Materials

The following supporting information can be downloaded at: https://www.mdpi.com/article/10.3390/nano13172467/s1.
