# Supplementary material for: Facile Transfer of Spray-Coated Ultrathin AgNWs Composite onto the Skin for Electrophysiological Sensors
Source: Nanomaterials (Basel). 2023 Aug 31;13(17):2467. doi: 10.3390/nano13172467 (PMC10489915; doi:10.3390/nano13172467)
Supplement: Supplementary file 1 [file nanomaterials-13-02467-s001.zip › nanomaterials-2565385-supplementary.pdf]

nanomaterials

Supplementary Materials

Facile Transfer of Spray-Coated Ultrathin AgNWs Composite onto the Skin for Electrophysiological Sensors

Minwoo Lee †, Jaeseong Kim †, Myat Thet Khine, Sunkook Kim * and Srinivas Gandla *

Multifunctional Nano Bio Electronics Lab, Department of Advanced Materials Science and Engineering, Sungkyunkwan University, Suwon 16419, Republic of Korea; alchemist@skku.edu (M.L.); j2castle@naver.com (J.K.); myatthetkhine0997@gmail.com (M.T.K.)
*Correspondence: seonkuk@skku.edu (S.K.); srinivas@skku.edu (S.G.)
† These authors contributed equally to this work.

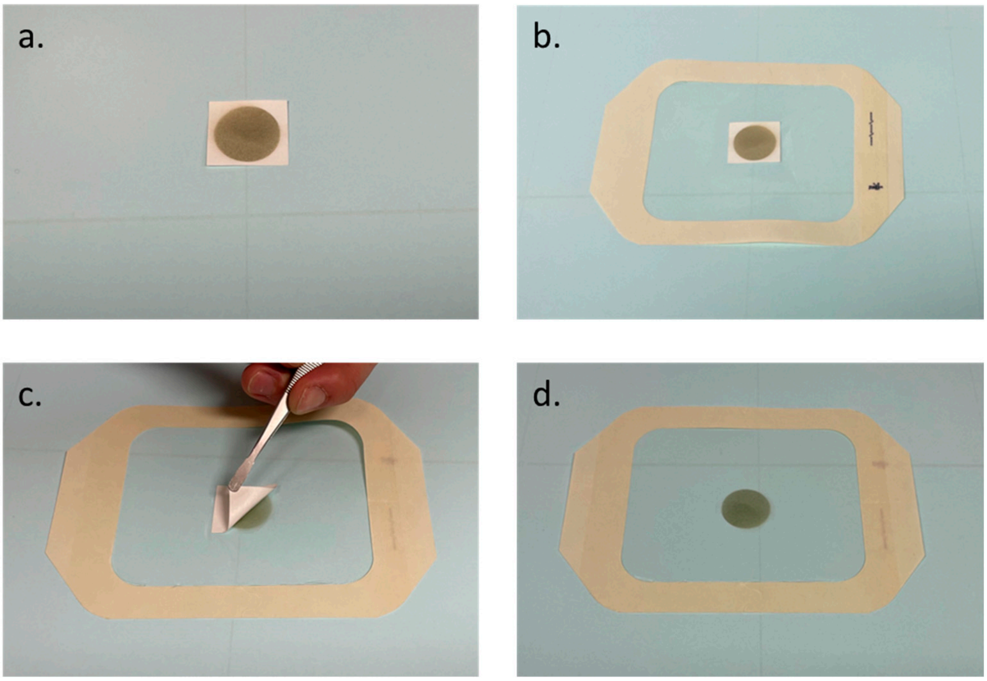

Figure S1. Transfer of AgNWs-based electrode to Tegaderm tape. (a) AgNWs on glossy paper, (b) placing Tegaderm on AgNWs, (c) removal of glossy paper, and (d) complete transfer of AgNWs to Tegaderm.

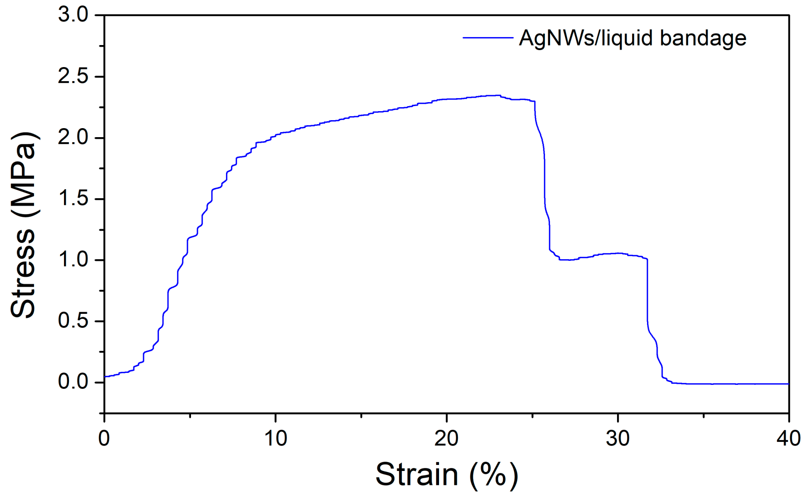

Figure S2. Stress-strain curve of the liquid bandage supported AgNWs-based film; film length, width, and thickness are 7 mm, 9 mm, and 3 μm. The film has a Young's modulus of ~0.45 MPa in the linear region.

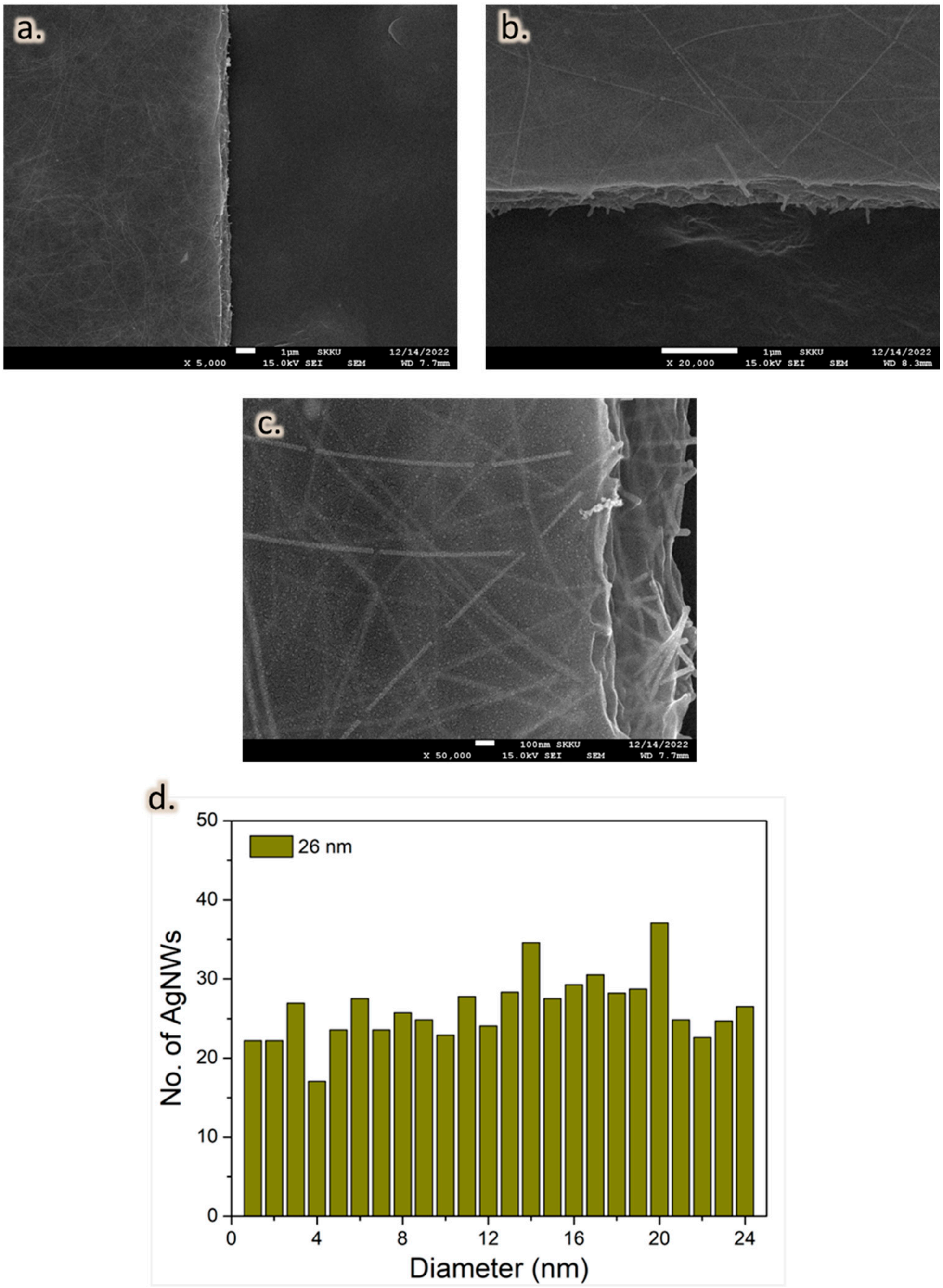

Figure S3. (a-c) SEM micrographs of the spray-coated AgNWs and hydroxypropyl methylcellulose composite solution, at different magnifications and angles. (d) The Distribution of the number of AgNWs by diameter (mean of 26.3 nm).

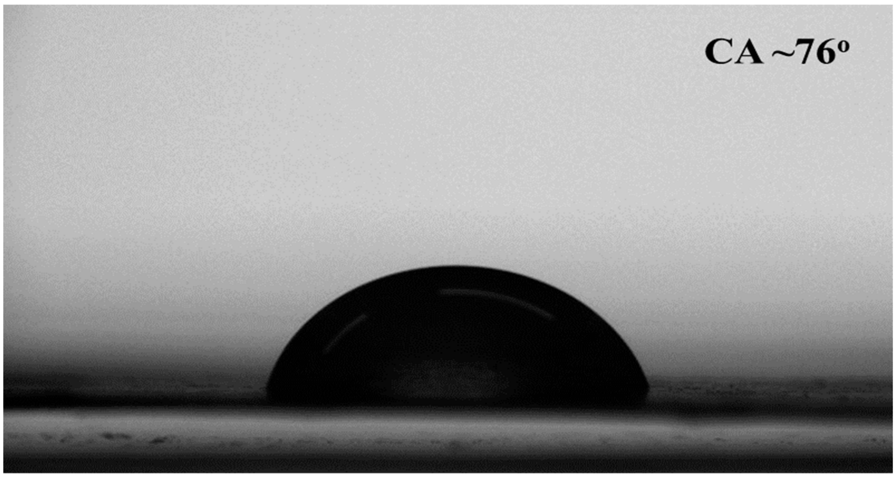

Figure S4. CA of DI water droplet on glossy paper.

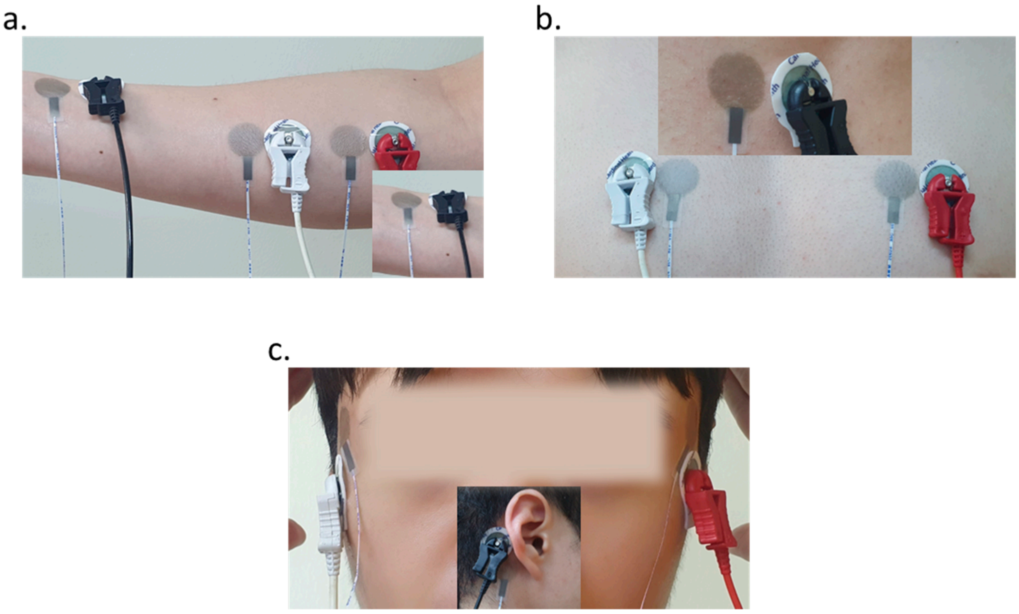

Figure S5. Conventional and AgNWs-based electrodes position on the human skin. (a) EMG, (b) ECG, and (c) EOG.

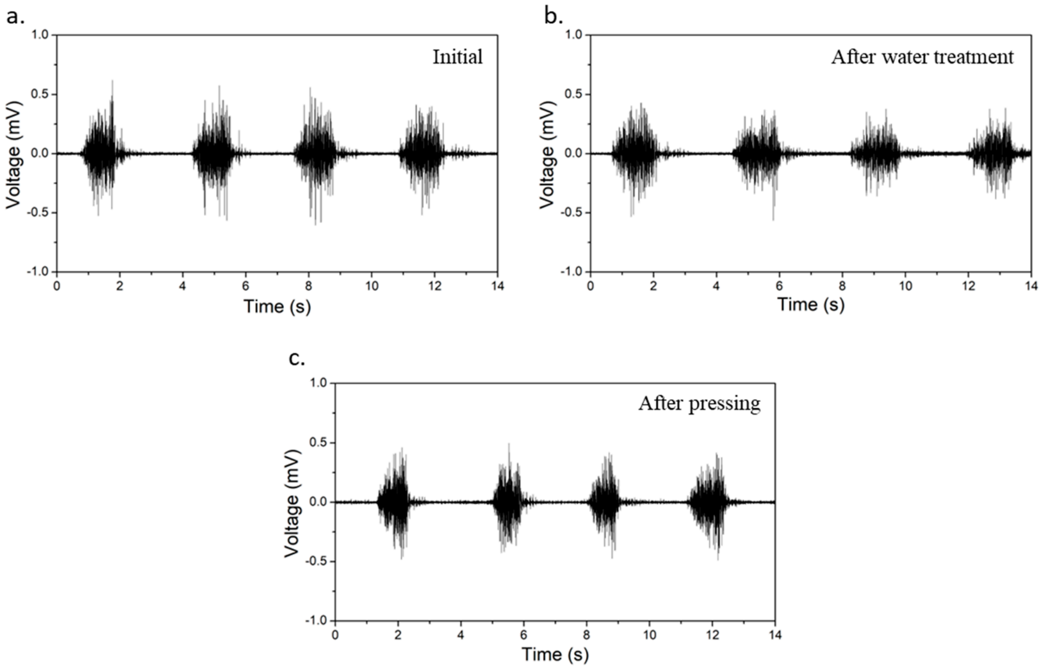

Figure S6. EMG signals measured before and after water treatment and pressing. (a) Initial, (b) after water treatment, and (c) after pressing.

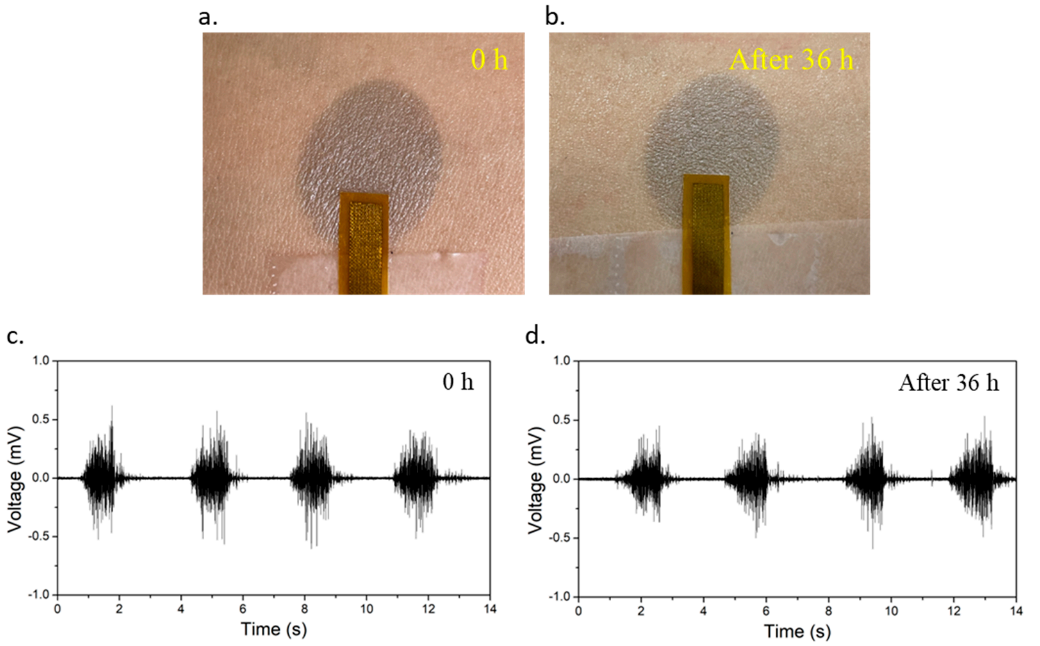

Figure S7. Long-term use of wearable AgNWs based sensor. (a,b) Photographed images of the sensor worn onto the skin captured (a) at 0 h and (b) after 36 h, with their corresponding measured (c,d) EMG signals.

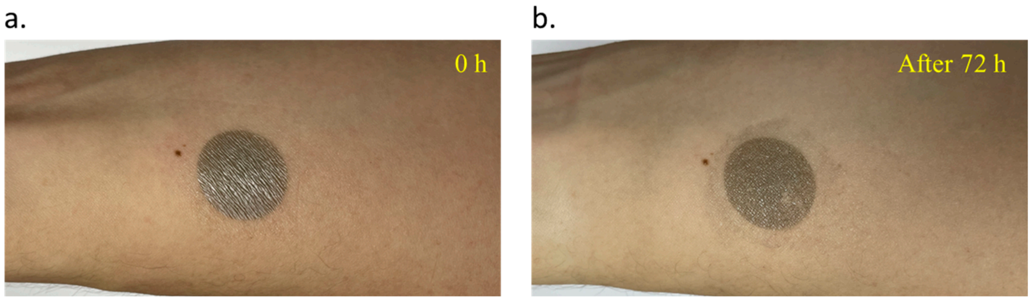

Figure S8. Wearable AgNWs-based sensor compatibility test with the skin. (a) 0 h and (b) after 72 h.

Table S1. Comparison of our work with previously reported research.

Electronic device	Adhesive materials	Transfer method	Transfer process of active materials [Direct/Indirect]	Active materials process	Ref.
TENG	3M Nexcare Liquid Bandage	Spraying	Indirect (Graphene on Cu foil → etching Cu → PET → skin)	Graphene (CVD)	[1]
EMG, ECG, and EOG	3M Tegaderm	Taping	Indirect (Graphene on Cu foil → etching Cu → tattoo paper → skin)	Graphene (CVD)	[2]
EMG and ECG	3M Nexcare Liquid Bandage	Spraying	Direct (AgNWs on porous TPU → skin)	AgNWs (dip-coating)	[3]
ECG	Water	Water drop	Direct (Au/Cr on PET → skin)	Au/Cr (thermal evaporation)	[4]
EMG and ECG	3M Nexcare Liquid Bandage	Spraying	Indirect (Si nanomembrane on SOI → elastomeric stamp or PVA → skin)	Si Nanomembrane (diffusion dopping on SOI wafer)	[5]
EMG and ECG	Water	Water drop (transferred with tattoo substrate)	Direct (PEDOT:PSS on tattoo paper → skin)	PEDOT:PSS (inkjet printing)	[6]
TENG	3M Nexcare Liquid Bandage	Spraying	Indirect (PI/Cu/PI on PMMA → PMMA removal → PI/Cu/PI on water-soluble tape → skin)	Cu (sputtering)	[7]
EMG, ECG, and EOG	3M Nexcare Liquid Bandage	Spraying	Direct (AgNWs on glossy paper → skin)	AgNWs (spraying)	This work

References

1. Chu, H.; Jang, H.; Lee, Y.; Chae, Y.; Ahn, J.H. Conformal, Graphene-Based Triboelectric Nanogenerator for Self-Powered Wearable Electronics. *Nano Energy* **2016**, *27*, 298–305, doi:10.1016/j.nanoen.2016.07.009.
2. Kireev, D.; Ameri, S.K.; Nederveld, A.; Kampfe, J.; Jang, H.; Lu, N.; Akinwande, D. Fabrication, Characterization and Applications of Graphene Electronic Tattoos. *Nat. Protoc.* **2021**, *16*, 2395–2417, doi:10.1038/s41596-020-00489-8.
3. Zhou, W.; Yao, S.; Wang, H.; Du, Q.; Ma, Y.; Zhu, Y. Gas-Permeable, Ultrathin, Stretchable Epidermal Electronics with Porous Electrodes. *ACS Nano* **2020**, *14*, 5798–5805, doi:10.1021/acsnano.0c00906.
4. Wang, Y.; Qiu, Y.; Ameri, S.K.; Jang, H.; Dai, Z.; Huang, Y.; Lu, N. Low-Cost, Mm-Thick, Tape-Free Electronic Tattoo Sensors with Minimized Motion and Sweat Artifacts. *npj Flex. Electron.* **2018**, *2*, 1–7.

5. Yeo, W.-H.; Kim, Y.-S.; Lee, J.; Ameen, A.; Shi, L.; Li, M.; Wang, S.; Ma, R.; Jin, S.H.; Kang, Z.; et al. Multifunctional Epidermal Electronics Printed Directly Onto the Skin. *Adv. Mater.* **2013**, *25*, 2773–2778, doi:10.1002/adma.201204426.
6. Taccola, S.; Poliziani, A.; Santonocito, D.; Mondini, A.; Denk, C.; Ide, A.N.; Oberparleiter, M.; Greco, F.; Mattoli, V. Toward the Use of Temporary Tattoo Electrodes for Impedancemetric Respiration Monitoring and Other Electrophysiological Recordings on Skin. *Sensors (Switzerland)* **2021**, *21*, 1–17, doi:10.3390/s21041197.
7. Wong, T.H.; Liu, Y.; Li, J.; Yao, K.; Liu, S.; Yiu, C.K.; Huang, X.; Wu, M.; Park, W.; Zhou, J.; et al. Triboelectric Nanogenerator Tattoos Enabled by Epidermal Electronic Technologies. *Adv. Funct. Mater.* **2022**, *32*, 1–10, doi:10.1002/adfm.202111269.
